# Engineering phytosterol-based oleogels for potential application as sustainable petrolatum replacement†

**DOI:** 10.1039/c9ra06950j

**Published:** 2019-12-24

**Authors:** Xiao-Wei Chen, Shang-De Sun, Guo-Long Yang, Chuan-Guo Ma

**Affiliations:** Lipid Technology and Engineering, School of Food Science and Engineering, Henan University of Technology Lianhua Road 100 Zhengzhou 450001 Henan PR China chenxiaowei8252@hotmail.com mcg@haut.edu.cn +86-371-67758022 +86-371-67758022; National Engineering Laboratory for Wheat & Corn Further Processing, Henan University of Technology Lianhua Road 100 Zhengzhou 450001 Henan PR China

## Abstract

Phytosterol-based oleogels have been engineered in edible oils for potential applications as sustainable replacements for petrolatum. Oleogels have emerged with a crystal network structure with oil molecules trapped inside. In addition, the viscosity of highly thixotropic oleogels could be tuned by manipulating the concentration of phytosterols and monoglycerides, and the type of surface-active small molecules and bulk vegetable oils. Furthermore, viscous soft matter could also be tunably made with 8–20% oleogelators in olive oil with favourable water vapour occlusive and wettability properties, in addition to having good texture, and outstanding thixotropic and thermal reversibility properties. These properties are quite similar to those of commercial petrolatum. This work demonstrates that the natural phytosterol-oleogels in edible oils can be a novel source of sustainable and green replacements for petrolatum.

## Abstract 2

Phytosterols, as a family of plant-produced molecules, are recognized for their natural health benefits. Phytosterol-based oleogels have been engineered in edible oils to be potential applicable as sustainable petrolatum replacements.

## Graphical abstract

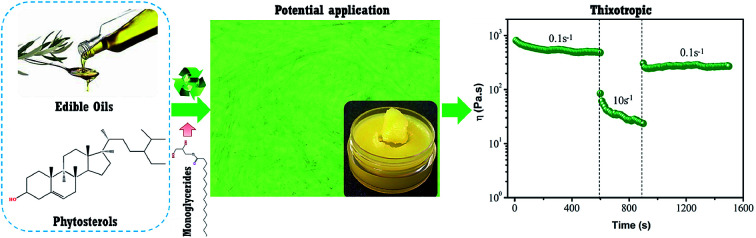

## 1. Introduction

Health and safety are priorities in the food and cosmetics industries. Unfortunately, current semi-occlusive technologies used to protect the skin barrier mostly involve petrolatum, which is derived from petroleum and has been identified as a potentially carcinogenic ingredient containing polycyclic aromatic hydrocarbons (PAHS).^1^ In addition, petrolatum can be perceived to have negative sensory properties for human health and the environment, and potential replacements from sustainable and priority resources have attracted a lot of attention. Petrolatum softens upon application and forms a water-repellent film around the applied area, creating an effective barrier against the evaporation of the skin's natural moisture, and it is a common moisturizer often used in the prevention of skin infections and in personal care products and cosmetics. Therefore, thixotropic behavior and recovery of viscosity and functionality are the important properties in a replacement product.^2,3^

Oleogels, as a new class of semi-solid functional material, have shown great promise for use in food and pharmaceutical industries, and those based on personal care products.^4^ They are promising because they offer desirable properties such as required structural properties, maintaining product spreadability and providing long-term stability.^4,5^ Typically, oleogelation can be achieved by the self-assembly of low molecular-mass oleogelators (LMOGs) to structure liquid oils in a three-dimensional network, such as waxes, 12-hydroxystearic acid, fatty acids or alcohols, phytosterols and their mixtures in vegetable oils.^6^ The liquid oil is stabilized due to the formation of physical bonds, such as van der Waals, π–π stacking and H-bonds, resulting in permanent rigid networks and semiflexible solid-like gels.^7,8^ Among the various oleogelators available, phytosterols, a family of plant-produced molecules, stand out because they are natural and healthy. More importantly, phytosterols have a similar chemical structure to cholesterol, which has positive health implication for the phospholipid membrane in cell structures.^9,10^

Generally, molecular engineering concepts could be applied to design and modulate the physicochemical and functional properties of oleogels, enabling functionality-targeting for specific applications. The addition of surfactants to oleogel systems has been identified as an available strategy to modify their microstructure and physical properties, such as their appearance, mechanical strength and plasticity.^11–13^ More recently, Sintang *et al.*, found that a combination oleogel of phytosterols and monoglyceride was formed with a bilayer structure, in which monoglyceride acts as an emulsifier that influences crystallization.^14,15^ Furthermore, we also found that phytosterol-based oleogels were self-assembled with monoglycerides *via* hydrogen bond interactions into a mixed crystal system that has enhanced physical stability and functional characteristics.^16,17^ From an environmental perspective, edible natural plant-oil structures are increasingly being taken into account as promising eco-friendly alternatives to replace mineral oil.^6,18^ Therefore, engineering phytosterol-based oleogels in edible oils could be a commercially and technologically viable route for petrolatum replacement.

The present work aims to engineer phytosterol-based oleogels in edible oils for the sustainable replacement of petrolatum. The effects of the concentration and weight ratio of oleogelators, and oil type on the thixotropic properties were evaluated. The oleogels were also characterized using water vapour permeability and wettability properties, compared to commercial petrolatum. In particular, the mechanical and thermal reversibility properties of oleogels were also studied by performing a small-deformation rheological test.

## 2. Materials and methods

### 2.1. Materials

Phytosterols (containing 82.44% β-sitosterol, 10.02% β-sitostanol, 3.32% campesterol, and 4.21% of other minor sterols, confirmed by GC analysis) were purchased from Xi'an Realin Biotechnology Co., Ltd (Xi'an, China).^17^ γ-Oryzanol was also purchased from Xi'an Realin Biotechnology Co., Ltd (Xi'an, China). Glycerol monooleate (≥50.0%), glycerol monostearate (>60.0%) and soy lecithin (>90%) were purchased from Aladdin (Shanghai, China). Commercial petrolatum jelly (SUQIE Vaseline Original Petroleum Jelly) was purchased from Guangzhou Sucui Cosmetics Co., Ltd. Edible oils (*e.g.*, sunflower oil, olive oil, soybean oil, and corn oil) with different fatty acid compositions (as seen in Table S of ESI†) were purchased from a local grocery market. Algae oil, containing a total omega-3 PUFA content ≥40% (as seen in Table S of ESI†) according to the manufacturer's specifications, was supplied by Runke Bioengineering Co., Ltd (China). All other chemicals were reagent grade and used as received.

### 2.2. Manufacture of oleogel formulations

Oleogel samples were prepared in 20 mL Pyrex beakers in 10 g batches. The standard oleogel treatments consisted of 10 wt% oleogelators and 90 wt% vegetable oils according to the process described in our previous work.^16^ For example a formula was made with 6 wt% phytosterols and 4 wt% monoglycerides in olive oil. The ingredients were heated to 85 ± 2 °C under magnetic stirring at 250 rpm for 30 min. Then, the clear oily dispersions were cooled down to 25 °C for 24 h prior to tests.

### 2.3. Steady shear measurements

The steady shear was measured using a HAAKE MARS60 rheometer (Thermo Fisher Scientific Inc., USA) equipped with a water bath (MultiTemp III; Amersham Biosciences, Little Chalfont, UK) for temperature control. After 24 h of storage at 25 °C the samples were slowly sheared by hand in their vials for 15 rotations clockwise and 15 rotations counter clockwise.^18^ A 35 mm 1° cone plate was lowered into the sample with a gap size of 1000 μm and the temperature was set to equilibrate to 25 °C for 5 min before measurement. A steady state flow test with a large shear rate ramping from 0 to 200 s^−1^ with 150 measurement points was used to measure the initial viscosity. The sample was then placed in a beaker for storage, and measured by replication after 1 week. The flow behavior index of the oleogels was calculated by fitting the experimental data to the power law model: *σ* = *kγ̇*^*n*^, where *σ* = shear stress (Pa), *γ̇* = shear rate (s^−1^), *n* = flow behavior index and *k* = consistency coefficient (Pa s).

### 2.4. Oscillatory measurements

Dynamic-oscillation measurements of the oleogels were carried out with a HAAKE MARS60 rheometer (Thermo Fisher Scientific Inc., USA). A cone plate (1°) geometry of 35 mm in diameter was used, and the geometric gap was set at 1000 μm. The sample was poured onto the plate and left for a resting period of 5 min to achieve thermal equilibrium before the experiments. The stress sweep was performed at 25 °C within the range 0.1–1000 Pa and with a fixed frequency of 1 Hz. For a small-deformation thixotropic evaluation, samples were subjected to time sweeps at alternating intervals of low and high shear rates with subsequent steps of 0.1 s^−1^ for 10 min and 10 s^−1^ for 1 min followed again by a shear rate of 0.1 s^−1^ for 10 min. Temperature sweeps, including heating from 5 °C to 80 °C, and then cooling to 5 °C at a rate of 5 °C min^−1^, were also carried out with a constant stress of 1.0 Pa and at a frequency of 1 Hz within the linear viscoelastic regime.

### 2.5. Microstructure observations

Optical and confocal microscopy techniques were utilized to study the microstructure of the samples. Optical microscopy was undertaken on an inverted U-HGLGPS microscope (Olympus, Tokyo, Japan). For confocal microscopy, Nile Red was first dissolved in oil, and this oil was then used to prepare the samples. Samples were imaged using a Leica confocal laser scanning microscope (CLSM, Leica Microsystems Inc., Heidelberg, Germany). The samples were examined using an argon krypton laser (ArKr, 488 nm).

### 2.6. Water vapour transmission rate

The water vapour transmission rate (WVTR) was measured using a previously described method.^19^ A mixture of 3% hydroxypropyl methyl cellulose (Sigma-Aldrich, Shanghai, China), 37.5% silica (Sigma-Aldrich, Davisil Grade 633, 60 Å, 200–425 mesh), 13.2% MgCl_2_·6H_2_O (Aladdin, Shanghai, China), and 46.3% water was prepared. 35 mm Petri dishes were used as sample holders and were filled with 50 g of the mixture, leaving an approximately 2 mm gap at the top of the dishes. The dishes were then placed in a freezer at −18 °C for 24 h. Oleogel samples were spread onto the dishes to fill the remaining space in the dishes. The samples were placed in a sealed desiccator with a saturated solution of MgCl_2_·6H_2_O in the bottom to control the relative humidity at 32.9%. The desiccator was placed in an incubator at 20 °C and the weight change of the samples was measured over time (6 days). A WVTR value was calculated using the formula described below [eqn (1)]:1
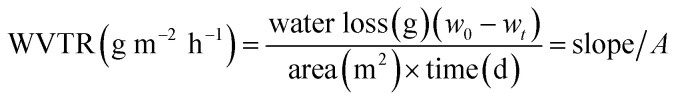


The area of the membrane was 38.465 × 10^−4^ m^2^; *w*_0_ and *w*_*t*_ were the weights in grams at the original and time (*t*) points, respectively. The slope is the slope of the linear portion of the weight loss over time (g per day) plot. Petrolatum was compared as a control. Samples were measured at least in triplicate.

### 2.7. Contact angles of water droplets on oleogel substrates

Contact angle measurements were conducted with a sessile drop method using an optical drop shape analyzer (OCA-20, Data-physics Instruments GmbH, Germany) at room temperature and with 18.2 ΩM water as the probe liquid.^17^ Slides were dipped into the hot oleogel solutions and then left at room temperature (25 ± 2 °C) for 24 h for gelation to occur. Contact angle (*θ*) measurements were taken exactly 60 s after placement of 5 μL droplets on the surface. The reported values were the averages of seven consecutive measurements for each sample.

### 2.8. Statistical methods

All data are reported as the mean ± standard deviation from duplicates or triplicates, and analyzed using SPSS 19.0 software. A one-way ANOVA Duncan's test was used to compare the significant difference in mean values at a *p* level of 0.05.

## 3. Results and discussion

### 3.1. Oleogelation

**Fig. 1:**
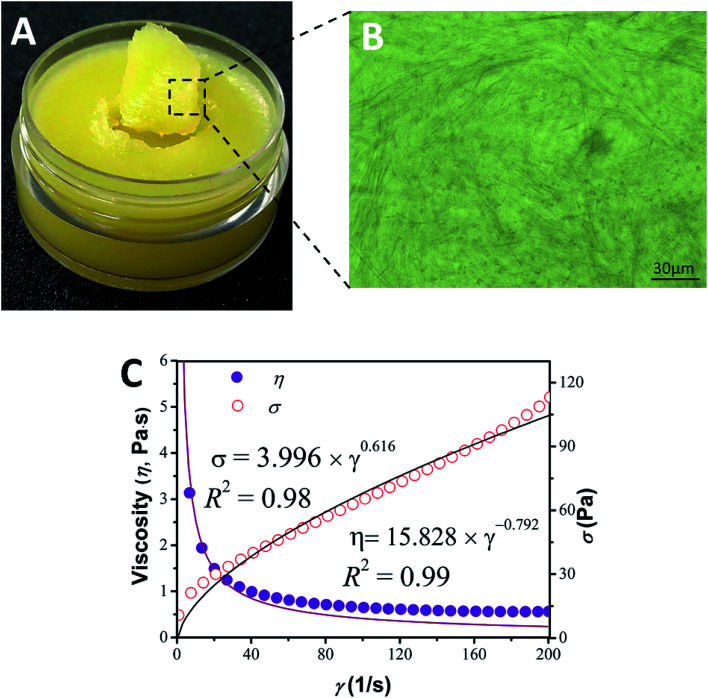
Appearance (A), optical microscopy (B), viscosity and shear stress with power law fit (C) of a sample made with 10 wt% phytosterol and monoglyceride in olive oil at the ratio of 6 : 4 (w/w).

Phytosterols are a family of compounds consumed by the human body as part of the diet, and are well known for their natural healthiness and biocompatibility in cosmetics. Moreover, apart from reducing serum cholesterol, phytosterols can repair a damaged skin barrier and keep the skin hydrated.^8^ Unfortunately, the oleogelation of phytosterols in triglyceride oil with large blade-shaped crystals was unstable.^14,16^ When subjected to shear after setting, the gel irreversibly breaks down, causing loss of its functionality, which seriously limits its application.^20^ In our recent research, phytosterols are in fact able to self-assemble with monoglyceride (one type of food-grade surfactant).^16^ resulting in a three dimensional crystal network in bulk oil, forming oleogels. Upon heating and cooling, the phytosterol molecules modified by monoglyceride were prone to conversion into a new needle-like crystal network in liquid oil, sequentially forming networks, and thus converting the liquid into a thixotropic oleogel, thick in texture and satiny in appearance (as shown in Fig. 1A and B). Considering that the raw materials were naturally healthy and biocompatible, the appearance, texture and thickness of the gel indicated that the phytosterol-based oleogel has good potential for application as a replacement for petrolatum from a visual perspective. We could also observe that the network microstructure of the synergistic oleogel was very similar to commercial petrolatum, which also undergoes gelation by forming needle-like crystal networks in mineral oil (as can be seen in Fig. S1 of ESI†). Systems perform several desirable functions which are related to their structure, implying that the phytosterol-based oleogel and commercial petrolatum have similar functional characteristics.^21^

As can be seen from Fig. 1C with a large shear rate ramping from 0 to 200 s^−1^, the power law model showed an excellent fit to the shear stress data with *R*^2^ = 0.98 and gave a value of *n* = 0.616 (<1) indicating greater shear thinning behavior compared to that found in a commercial petrolatum product (*n* = 0.839 < 1) (as can be seen in Fig. S2 of ESI†). Additionally, thixotropic behavior and recovery of viscosity and functionality are important properties in a replacement product. Therefore, to achieve an adequate thixotropic property for oleogels and provide useful information for the development of a good replacement for petrolatum, numerous factors would need to be controlled. The mechanical properties of oleogels could be manipulated by adjusting a variety of parameters, such as the type of triglyceride oil used, the concentration of phytosterols and monoglyceride employed, as well as by the addition of various surfactants.

### 3.2. Effect of monoglyceride concentration

**Fig. 2:**
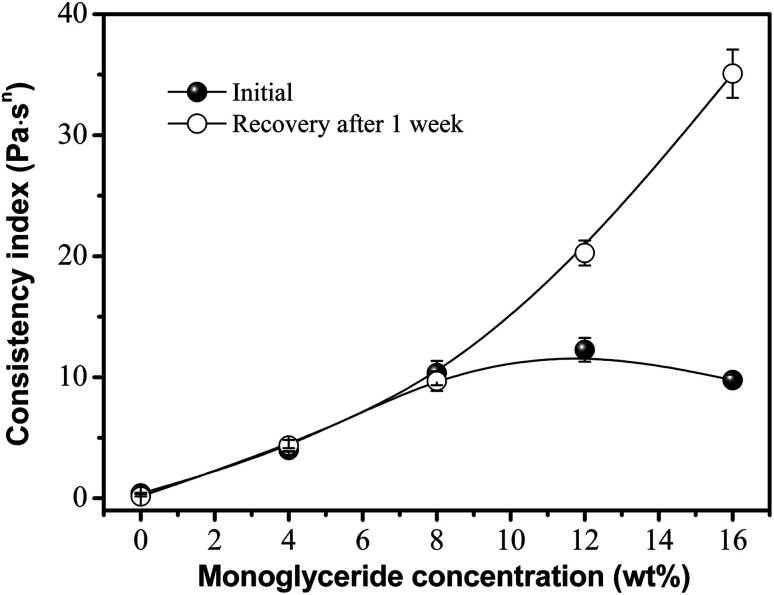
Effect of monoglyceride concentrations on the viscosity and recovery of oleogels made with 6 wt% phytosterols.

The rheological manifestation of viscosity-induced structural changes is reversible and time-dependent, and is called thixotropy. It is generally accepted that thixotropy is the phenomenon whereby a fluid shows a reversible structural transition and the assembly of oleogelators. Thus, thixotropy has been described as a material becoming less viscous under stress and returning to the initial state when the stress is removed.^21,22^ Thus, the consistency coefficient (*k*) over the time needed for the structure to regroup during shear or at rest can be used throughout our analyses, as an indicator of thixotropy. The value for *k* was obtained from the power law fit and plotted against the concentration of glycerol monostearate in the samples, as shown in Fig. 2. As the concentration of monoglyceride increased, the viscosity (from the consistency coefficient) of the gels increased. This possibly indicates a more cooperative process in which an extensive mesh network is formed by hydrogen bond interactions that enhances the viscosity of the oleogels.^14,15^ Consequently, the shapes of the phytosterol crystals in the mixed component systems varied significantly with the proportion of monoglyceride, as in our previous reports.^16^ This was expected since the more monoglyceride there is in the system, the more phytosterols junction zones can be contributed to the combination, increasing network strength, and resulting in a more needle-like crystal network and the better physical stability of the oleogels.^14–16^ However, further addition led to an excess of these molecules (*e.g.*, 16%) with a negative effect on the crystal network, thus decreasing the gel strength. It was observed that the proportion of viscosity recovered after one week of storage was close to 100% for concentrations below 8%, but increased to around 200% and 350% for monoglyceride concentrations of 12 and 16%, respectively. As we know, this time-dependent behavior can be attributed to the disarrangement–rearrangement processes of the crystal network along the direction of shear. Thus, we suggest that this increase in recovery is the result of the rearrangement of the hydrogen-bonding network supporting the oleogels, that leads to a more efficient gel net after one week, based on previous work by Sintang *et al.*^14^ Moreover, the variation among the monoglyceride concentrations is perhaps related to an amphiphilic characteristic. A higher monoglyceride concentration would modify the phytosterol crystals into triglyceride oil by hydrogen bonding interaction. The result was in agreement with previous research by both our laboratory and Bin Sintang *et al.*, where the monoglyceride affected the microstructural development of the phytosterol-based oleogels, forming a mixed crystal system.^14–16,23^ Moreover, this promotes the gels' ability to reassemble at all the bond points that were present in the initial sample, resulting in a higher viscosity after one week of recovery. Expectedly, at a lower monoglyceride concentration (<4%), oleogels were still formed; however, they were much weaker. It is thought that the phytosterols were not solubilized enough to produce a thixotropic gel and sediment during storage. Thus, in the case of lower monoglyceride concentrations, the oleogels appeared to be broken down during the pre-shear procedure and had very low viscosity and recovery.

### 3.3. Effect of phytosterol concentration

**Fig. 3:**
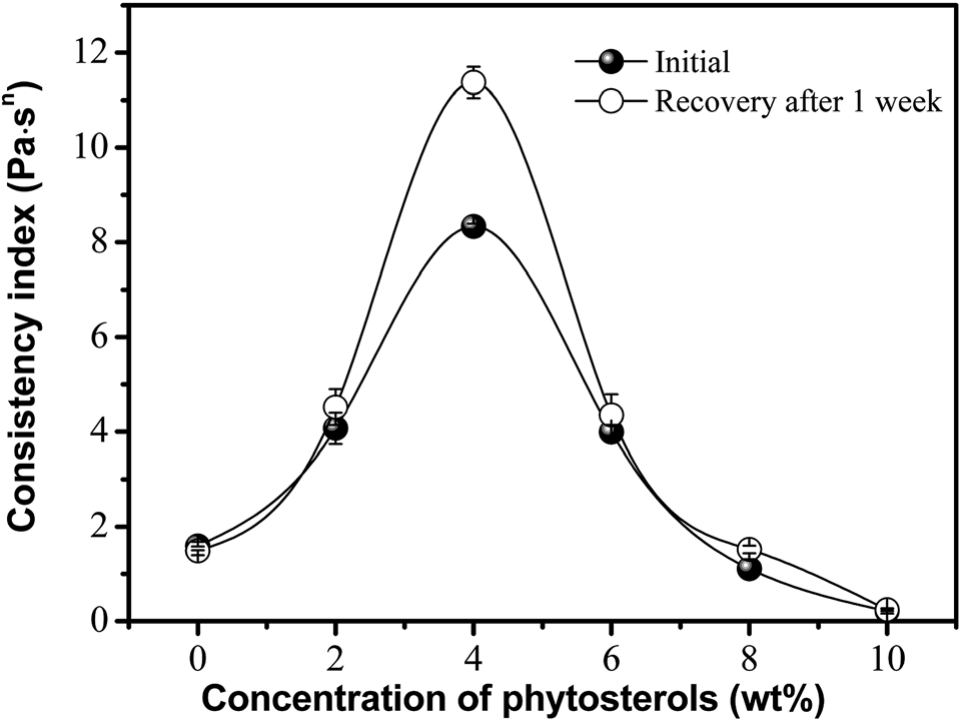
Effect of phytosterol concentration on the viscosity and recovery of oleogels made with 4 wt% monoglyceride.

One significant factor was observed in phytosterol incorporated oleogels, as shown in Fig. 3. The greatest viscosity and recovery were achieved between phytosterol concentrations of 2% and 6%. Oleogels made with 4% phytosterols can be considered the most promising due to their high viscosity and thixotropic character. And oleogels made with low phytosterol concentration appeared to be broken down with a very low viscosity, which was mainly due to shear-induced structural breakdown. Actually, the oleogel formation is a result of the balance between solubility and insolubility of the gelator (*e.g.*, phytosterols, monoglyceride) within the solvent (*e.g.*, edible oil) and the solvent–gelator and gelator–gelator interactions.^23^ Higher phytosterol concentrations of above 6% may limit the capability to dissolve within the solvent and find neighbouring oleogelators with which to interact. This indicates that the extra hydroxyl of phytosterols is able to weaken the oleogel matrix. We also note that the proportion of consistency coefficient recovered after one week of storage was close to 140% in a concentration of 4%, but decreased to around 100% for phytosterol concentrations of <2% or >6%. Presumably, this is related to an assembly behavior caused by the intermolecular hydrogen bonds between phytosterols and monoglyceride, and thus they are ideally solubilized for gelation to occur.^15,16,24^

### 3.4. Effect of surfactant type

**Fig. 4:**
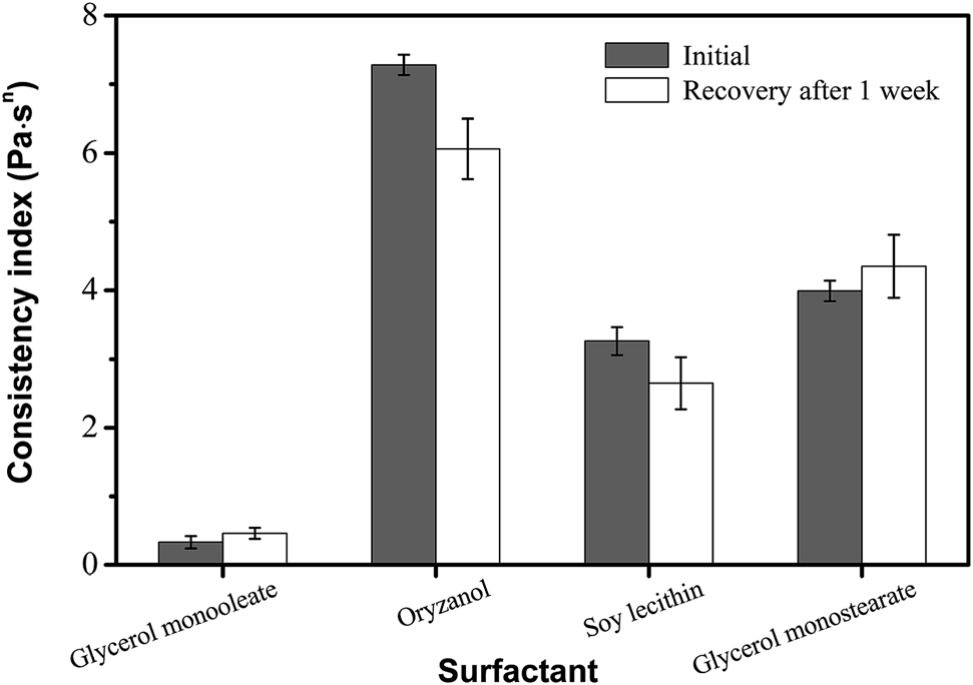
Effect of surfactant type on the viscosity and recovery of oleogels made with 6 wt% phytosterol and 4 wt% surfactant.

The effect of surfactants on the oleogels has been well documented in crystal growth behavior by synergistic interactions. This may provide one means to modify physicochemical and functional properties.^11,25,26^ Several surfactants (*e.g.*, glycerol monooleate, oryzanol, soy lecithin, and glycerol monostearate) were studied for their efficacy in engineering phytosterol-based oleogels, as shown in Fig. 4. Oryzanol showed a much higher initial consistency coefficient than glycerol monostearate but a reduced recovery of viscosity after one week of storage. Additionally, soy lecithin showed a much lower initial consistency coefficient than glycerol monostearate and it also had a reduced recovery of viscosity after one week. Previous work has shown that various surfactants have a significant effect on oleogel mechanical properties; in particular, a larger head group translates into a higher storage modulus.^5,27,28^ Furthermore, apart from their self-assembly and crystallization ability, surfactants' amphiphilic nature allows them to alter the solubilisation of the oleogelators in the oil phase.^16,29^ In this case, the phytosterols had better solubility in the sample made with lecithin, leading to a greater proportion of viscosity recovery compared to glycerol monooleate.^25,30^ However, the viscosity of the oleogel with lecithin showed less recovery back to the initial concentration than that of oleogel with glycerol monooleate after 1 week. This may be due to the stability of different oleogel systems. The oleogel developed by monoglyceride and phytosterols may structure oil by the well-known crystallization behaviour, resulting in a higher stability than that of an oleogel developed with lecithin and phytosterols.^14–16,23,25^ Furthermore, glycerol monostearate has a relatively large capability in both solubilization and self-assembly with phytosterols that leads to better gelation and a correspondingly high consistency index with high viscosity recovery.

### 3.5. Effect of oil type

**Fig. 5:**
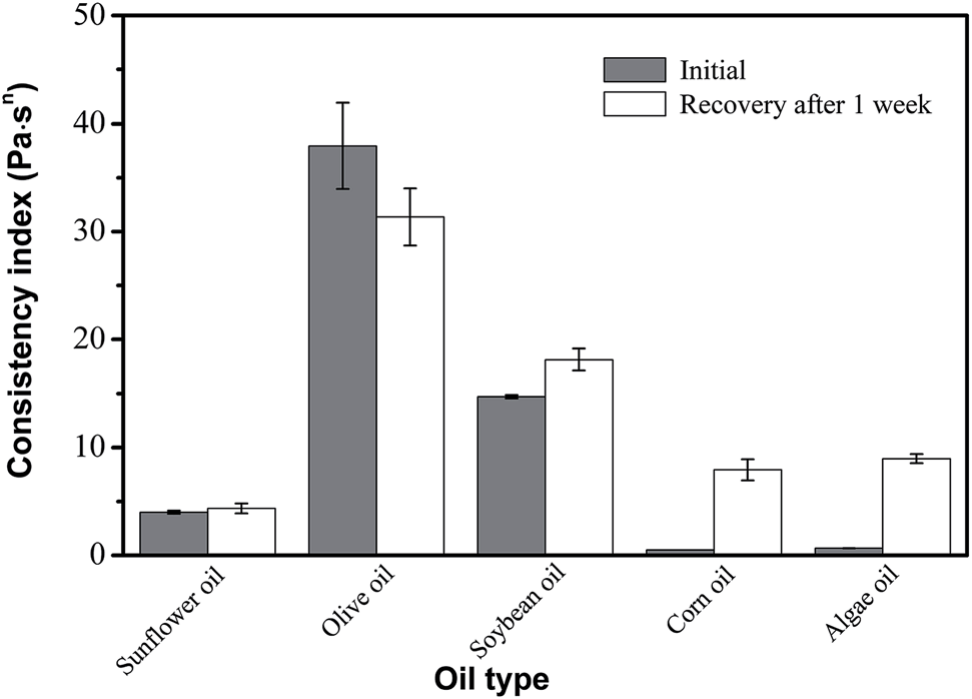
Effect of oil type on the viscosity and recovery of oleogels made with 6 wt% phytosterol and 4 wt% monoglyceride.

Several different types of oil were tested for their efficacy in creating desired oleogels. All oils tested showed similar results with obvious variations in viscosities and all oils were able to form thixotropic gels (Fig. 5). However, the degree of viscoelastic recovery of these oleogels is also greatly influenced by oil type. Oleogel made with olive oil showed the highest consistency index and recovery viscosity. Correspondingly, a gel made with sunflower oil showed a lower viscosity that was very close to its recovered viscosities. These observed variations in viscosity may be explained by variations in the polarity and degree of unsaturation of the oils tested, and the solvent–oleogelator compatibility was interpreted using Hansen's solubility theory.^3,31,32^ From these results, it can be seen that consistency index was positively correlated with solvent polarity when soybean oil was mixed with either sunflower oil or corn oil. Moreover, the properties of oleogel made with olive oil could be attributed to both oil polarity and the presence of minor components (polyphenol), enhancing the solubility of oleogelators, resulting in a higher consistency index. In previous studies Gravelle *et al.*^31^ and Okuro *et al.*^32^ also reported that oleogels' mechanical strength being positively correlated with solvent polarity could mostly be attributed to the ability of the polar entities present in the oil phase to interact with the oleogelators by forming hydrogen bonds.

### 3.6. Comparison to a commercial cosmetic product

#### 3.6.1 Water vapour transmission rate (WVTR) and wettability

**Fig. 6:**
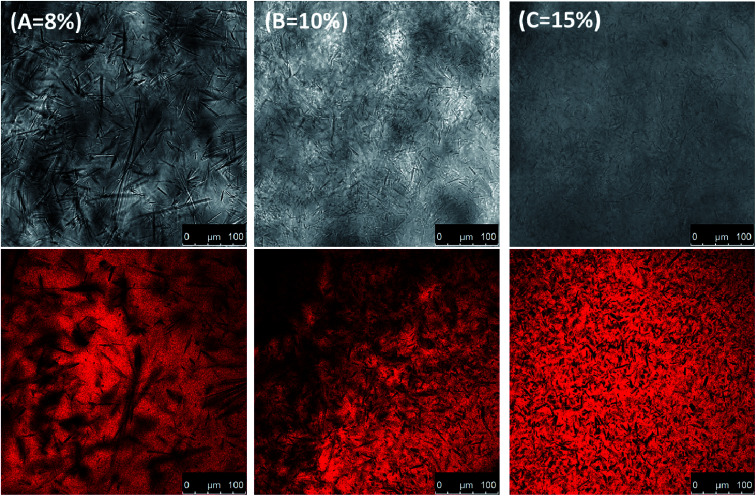
Light microscopy (LM) and confocal laser scanning microscopy (CLSM, oil were stained with Nile Red) images of the phytosterol and monoglyceride self-assembled oleogels by different oleogelators ((A) 8%, (B) 10% and (C) 15% with a phytosterol : monoglyceride ratio of 6 : 4 (w/w)) in the samples.

The capability of an occlusive material to control water loss can be determined by WVTR measurements. In general, the transport of water vapor is believed to be retarded due to the presence of a barrier layer. Indeed, the water vapour transmission rates of the covered gels were significantly superior to those of the uncovered control (49 × 10^−3^ g cm^−2^ d^−1^),^18^ as shown in Table 1. It was also found that the WVTR values of the gel samples were significantly (*p* < 0.05) higher than those of the ungelled oil. Although the hydroxyl groups were believed to have compatibility with water molecules, weakening the water vapor barrier properties, the high breathability of the oleogels may be attributed to the larger crystals having higher porosity compared to the pure system, which facilitated the mass transfer of small molecules.^16,24,33^ In the case of oleogel systems, the open space in a self-assembled crystalline network (*e.g.*, pores) could provide channels to increase the movement of small molecules within the crystals (see the light microscopy (LM) and confocal laser scanning microscopy (CLSM) images in Fig. 6A–C). Consequently, it can be observed that, as the amount of oleogelator increases, the WVTR becomes less occlusive (Table 1). However, compared to a commercially available commercial product, the tunable gels with varying co-oleogelator concentrations of 8–20% provide a protective occlusive barrier, though the commercial product did perform better under these test conditions.

**Table 1:** WVTR and water contact angles of oleogel samples compared to a commercial product

Samples	WVTR/g cm^−2^ d^−1^ (×10^−3^)	Water contact angle (°)
8% oleogel^a^	0.81 ± 0.05^ab^	72 ± 2^a^
15% oleogel^a^	1.6 ± 0.1^bc^	76.0 ± 0.5^a^
20% oleogel^a^	1.8 ± 0.2^c^	75 ± 1^a^
Ungelled olive oil	0.26 ± 0.04^a^	—^NA^
Control	0.602 ± 0.005^a^	87 ± 3^b^

a Phytosterol and monoglyceride self-assembled oleogels with different oleogelators (8%, 15% and 20% with a phytosterol : monoglyceride ratio of 6 : 4 (w/w)) in the samples. The results are shown as a mean with standard deviation for at least three samples. Values for different systems that have different small superscript letters differ significantly (*p* < 0.05). The control is commercial petroleum jelly. ^NA^ is not available.

It is known that the hydrophobicity or hydrophilicity of materials can reflect their surface properties;^34^ therefore, the water contact angle of the oleogels' surface was also determined to evaluate their wettability. The hydrophilic surfaces were generally related to the water contact angle and reflected their wettability. As can be seen from Table 1, all oleogels showed similar low water contact angle values (below 80°), which may be attributed to the rehydration of the surface. Besides increasing hydrophilicity of the sample, the oleogelator also changed both the morphological structure and the compactness with more small oleogel crystals (Fig. 6). As can be seen from Table 1, all oleogels showed similar low water contact angle values (below 80°), which may be attributed to rehydration of the surface. Besides increasing the hydrophilicity of the sample, the oleogelator also changed both the morphological structure and the roughness of the oleogels' surface (Fig. 6). The higher water contact angle of commercial petrolatum can be explained by the raw materials, which were mostly long-chain hydrocarbons from petroleum. This is in good agreement with the water vapour transmission rate.

#### 3.6.2 Rheological properties

**Fig. 7:**
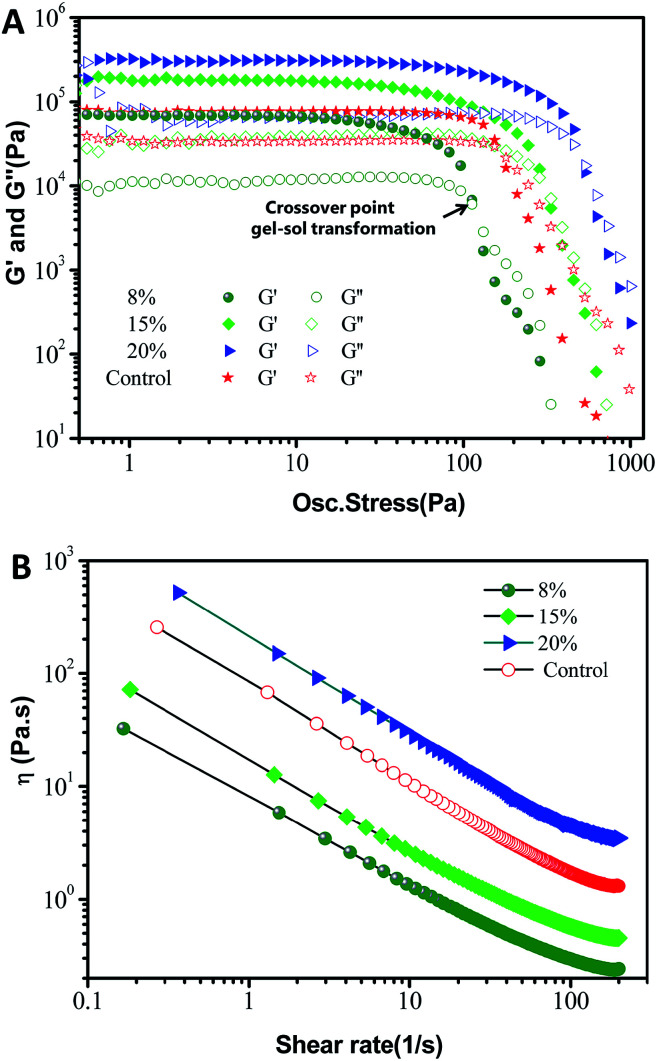
Storage modulus (*G*′) and loss modulus (*G*′′) from (A) amplitude stress sweeps, (B) viscous flow curves carried out on oleogel samples prepared with different oleogelators (8%, 15% and 20% with a phytosterol : monoglyceride ratio of 6 : 4 (w/w)) in the samples, with a control of petrolatum. The amplitude stress sweep was performed at 25 °C at a fixed frequency of 1 Hz.

From the appearance of the soft material shown in Fig. 1, the oleogels should reveal rheological behavior. Thus, to gain an insight into the mechanical properties, their rheological behavior was further studied through small-deformation amplitude stress sweeps, frequency sweeps and flow measurements (Fig. 7). As can be seen from the amplitude sweep curves of Fig. 7A, the value of the storage modulus (*G*′) was generally higher than those found for the loss modulus (*G*′′) in their individual linear viscoelastic regions (LVR) for both oleogels and the commercial petrolatum studied, suggesting that the gels mostly exhibit an elastic solid-like behavior. A marked increase in gel strength corresponded to an increase in oleogelator concentration from 8% to 20%, which revealed an elastic-dominant behavior, with a gradual increase in stiffness at high oleogelator concentrations. This is attributed to the liquid oil trapped in the crystal networks, which has a greater ability to resist external forces. As *G*′ decreases more quickly than *G*′′ with a further increase in stress amplitude, a crossover phenomenon (*G*′ = *G*′′) is observed with a gel–sol transition. At this degree of deformation (above the crossover point of the gel–sol transition), the oleogels show a more viscosity-dominant behavior. This effect may be due to the structural breakdown of the crystal network. Moreover, it can be observed that the critical stresses (*γ*_co_) of these oleogels increase significantly (*p* < 0.05) with an increase in the oleogelator concentration (Table 2), suggesting that the structure of the oleogels is mainly dependent on the crystal network. Furthermore, the storage moduli (*G*′) at the crossover point of these oleogels are also significantly (*p* < 0.05) increased (Table 2), indicating that the oleogels have greater strength at the critical collapse point with an increase in oleogelator. In addition, as can be seen from Fig. 7B, oleogels showed a strong shear-thinning flow behavior from the approximately straight lines with negative slopes that can be seen in the log–log plot. The viscosity values of phytosterol-based oleogel are similar to those obtained with the control sample (see Table 2), despite the similar low values of the flow index found in both systems, typical of yielding materials used as lubricating greases.^35^ Interestingly, these rheological indexes of a commercial petroleum jelly were located within the range of these oleogels, as shown in Table 2. These results further suggested that the phytosterol-based oleogels self-assembled with monoglyceride, resulting in a viscoelastic material and appeared to have greater spreadability.

**Table 2:** The consistency (*K*) and flow indexes (*n*) obtained from viscous flow behavior, crossover strain (*γ*_co_) and storage modulus (*G*′) at crossover strain obtained from strain sweep dynamic rheological measurements, structure recovery obtained from thixotropic rheological measurements and storage modulus (*G*′) at 37 °C obtained from thermoresponsive measurements of oleogel samples compared to commercial product

Samples	Flow behavior^c^			Amplitude sweep^d^		Thixotropic behavior, structural recovery	Thermoresponsive behavior, *G*′ (×10^3^ Pa) at 37 °C
	*K* (Pa s)	*n*	*R* ^2^	*γ*_co_	*G*′ (×10^3^ Pa) at co		
8% oleogel^a^	8.21 ± 0.03^d^	0.24 ± 0.01^a^	0.9995	112 ± 23^c^	6.8 ± 0.2^d^	37.3 ± 0.5%^b^	1.68 ± 0.31^c^
15% oleogel^a^	18.04 ± 0.08^c^	0.186 ± 0.005^b^	0.9992	287 ± 25^b^	15.9 ± 0.5^c^	54 ± 1%^a^	14.58 ± 3.25^b^
20% oleogel^a^	217.9 ± 0.4^a^	0.133 ± 0.007^c^	0.9997	458 ± 38^a^	47 ± 1^a^	50 ± 2%^a^	24.89 ± 0.93^a^
Control^b^	82.8 ± 0.2^b^	0.140 ± 0.004^c^	0.9998	154 ± 19^c^	35 ± 2^b^	31 ± 1%^c^	2.86 ± 0.27^c^

a Phytosterol and monoglyceride self-assembled oleogels with different oleogelators (8%, 15% and 20% with a phytosterol : monoglyceride ratio of 6 : 4 (w/w)) in the samples.

b The control is commercial petroleum jelly.

c The consistency (*K*) and flow indexes (*n*) obtained from viscous flow curves by a fairly good fit to the power-law model (*η* = *Kγ*^*n*−1^).^34^

d Co is the crossover strain point. The results are shown as a mean with standard deviation for duplicate samples. Values for different systems that have different small superscript letters differ significantly (*p* < 0.05).

**Fig. 8:**
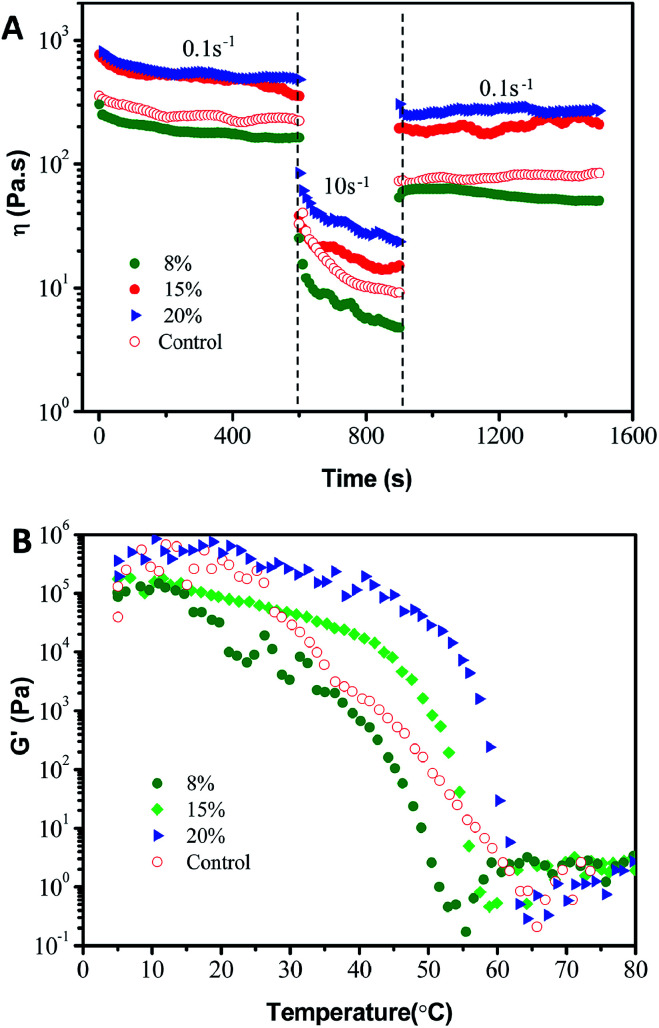
(A) Thixotropic behavior and (B) temperature ramp tests with a constant stress of 1.0 Pa and at a frequency of 1 Hz carried out on oleogel samples prepared with different oleogelators (8%, 15% and 20% with a phytosterol : monoglyceride ratio of 6 : 4 (w/w)) in the samples, with a control of petrolatum.

Additionally, the thixotropic behavior of the oleogels with small deformation was further studied to gain insights into their structure–recovery properties. The thixotropic recovery properties of the oleogels were evaluated *via* a three-interval time test (3-ITT), as shown in Fig. 8A. All the oleogels exhibited a progressive decrease in the storage modulus at a constant shear rate (0.1/10 s^−1^). However, as shown in Table 2, the 3-ITT experiment revealed that the oleogels showed a significantly (*p* < 0.05) higher structural recovery (from 37.3 ± 0.5% to 54 ± 1%) than that of commercial petroleum jelly (31 ± 1%) when the shear rate changed from 10 s^−1^ to 0.1 s^−1^, indicating comparable structural recovery and mechanical stability. The thixotropic behavior may be due to the lower recovery time for the reorganization of hydrogen bond forces between the hydroxyls in the soft systems, compared to the results of Fig. 2–5.^16,26^ This is very encouraging for potential sustainable applications to food-grade vaseline.^36^

To use oleogels for different types of applications, their response to temperature is also of interest. Theoretically, the oleogels should have an interesting thermoresponsive behavior based on the fact of the noncovalent cross-links between oleogelators. As expected in Fig. 8B, all of the oleogels showed thermo-responsive gelation during heating from 5 °C to 80 °C, suggesting a thermo-reversible physical characteristic. Simultaneously, petrolatum also showed a qualitatively similar trend with a general decrease in *G*′ with an increase in temperature. These observations are consistent with those visual perspectives and microstructures in Fig. 1 and S1.† Moreover, as can be observed in Table 2, the *G*′ value at 37 °C (the surface temperature of the human body) of these phytosterol-based oleogels clearly remained close to the value of commercial petroleum jelly (2.86 ± 0.27 kPa). Taken together, the commercial product and the oleogels from phytosterols and monoglycerides have similar rheological behavior, which further implies that oleogels have high viscoelasticity, consistency and the correct mechanical properties for a replacement for commercial petroleum jelly. Furthermore, these properties can also be tailored by modifying the oleogelator concentrations.

## 4. Conclusion

Phytosterol-based oleogels were successfully engineered in edible oils as a sustainable alternative to petrolatum. The phytosterol-based oleogels with monoglycerides were highlighted by a crystal network structure with liquid oil molecules trapped inside by hydrogen bond interactions. The viscosity and thixotropic property of the oleogels could be tailored by tuning the concentration of phytosterols and monoglycerides, the type of surfactants and the liquid oil phases. A particular combination of phytosterols and glycerol monostearate gave a superior product with high viscosity recovery, showing great potential in food, cosmetic and pharmaceutical applications. Furthermore, soft oleogels were developed that could be tuned to have favourable water vapour occlusive and wettability properties with a good texture and an outstanding thixotropic behavior, showing physical attributes similar to petrolatum. Taking the phytosterol activity into account, the oleogels were shown to be soft and comfortable for a natural and sustainable alternative to petroleum for food-grade Vaseline production.

## Conflicts of interest

There are no conflicts to declare.

## Supplementary Material

RA-010-C9RA06950J-s001
