# Supplementary material for: Engineering phytosterol-based oleogels for potential application as sustainable petrolatum replacement
Source: RSC Adv. 2019 Dec 24;10(1):244–52. doi: 10.1039/c9ra06950j (PMC9047561; doi:10.1039/c9ra06950j)
Supplement: RA-010-C9RA06950J-s001 [file RA-010-C9RA06950J-s001.pdf]

Electronic Supplementary Material (ESI) for RSC Advances.
This journal is © The Royal Society of Chemistry 2019

Supporting Information (SI)

Engineering Phytosterol-based Oleogels for Potential Application as Sustainable Petrolatum Replacement

Xiao-Wei Chen,[a]* Shang-De Sun,[a] Guo-Long Yang,[a] and Chuan-Guo Ma[ab]*

[a] Lipid Technology and Engineering, School of Food Science and Engineering, Henan University of Technology, Lianhua Road 100, Zhengzhou 450001, Henan Province, PR China

[b] National Engineering Laboratory for Wheat & Corn Further Processing, Henan University of Technology, Lianhua Road 100, Zhengzhou 450001, Henan Province, PR China

Running title: Engineering Phytosterol-based Oleogels in Edible Oils

Corresponding to:

Tel/fax: (086)371-67758022.

Xiao-Wei Chen:

E-mail address: chenxiaowei8252@hotmail.com; ORCID: 0000-0003-4564-5120

Chuan-Guo Ma:

E-mail address: mcg@haut.edu.cn (C.-G. Ma)

Table S. fatty acid combination of the edible oils (sunflower oil, olive oil, algae oil, corn oil, soybean oil) used in the work.

**Fatty acids**	**Olive oil**	**Algae oil**	**Soybean oil**	**Corn oil**	**Sunflower oil**
Myristic $C_{14:0}$	0.01±0.01	2.63±0.11	—[n.d.]	0.04±0.01	—
Palmitic $C_{16:0}$	13.45±0.34	37.13±0.52	10.5±0.1	13.28±0.82	6.17±0.00
Stearic $C_{18:0}$	2.23±0.22	1.29±0.03	4.5±0.1	1.75±0.33	3.51±0.01
Oleic $C_{18:1}$	73±1	0.59±0.01	56.47±0.81	31.21±0.0.32	27.53±0.35
Linoleic $C_{18:2}$	10.88±0.09	0.62±0.02	20.00±0.24	52.08±0.91	61.47±0.18
Linolenic $C_{18:3}$	0.89±0.04	0.31±0.02	7.7±0.1	1.2±0.1	—
Arachidic $C_{20:0}$	0.33±0.03	—	0.35±0.05	0.35±0.06	0.25±0.00
Eicosenoic $C_{20:1}$	0.28±0.01	—	0.24±0.03	—	0.19±0.00
Arachidonic $C_{20:4}$	—	0.09±0.01	—		—
Behenic $C_{22:0}$	0.08±0.01	—	0.34±0.00	—	0.49±0.37
DPA $C_{22:5}$	—	9.53±0.51	—	—	—
DHA$C_{22:6}$	—	41.27±1.23	—	—	—

Values are mean ± standard deviation. [n.d.] not detected (< LOD).

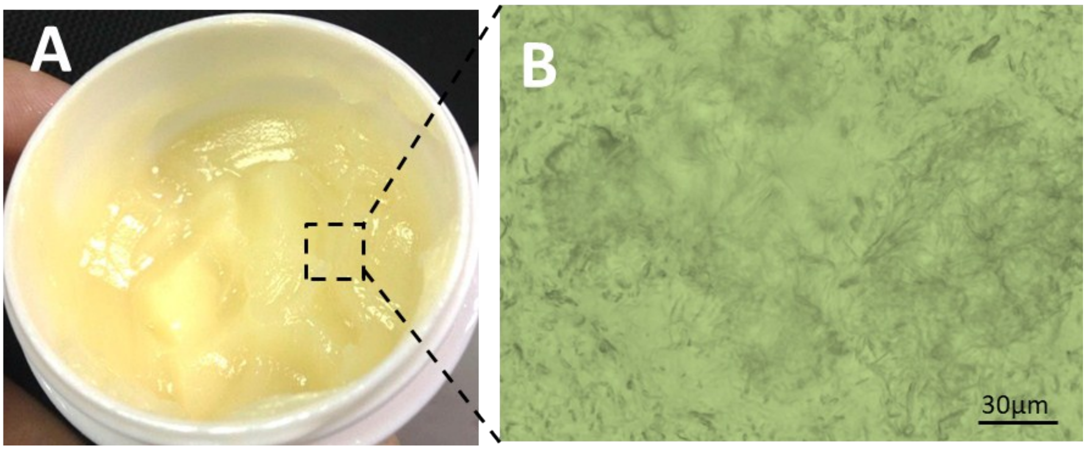

Fig. S1 Appearance (A) and optical microscopy (B) of a commercial petrolatum product.

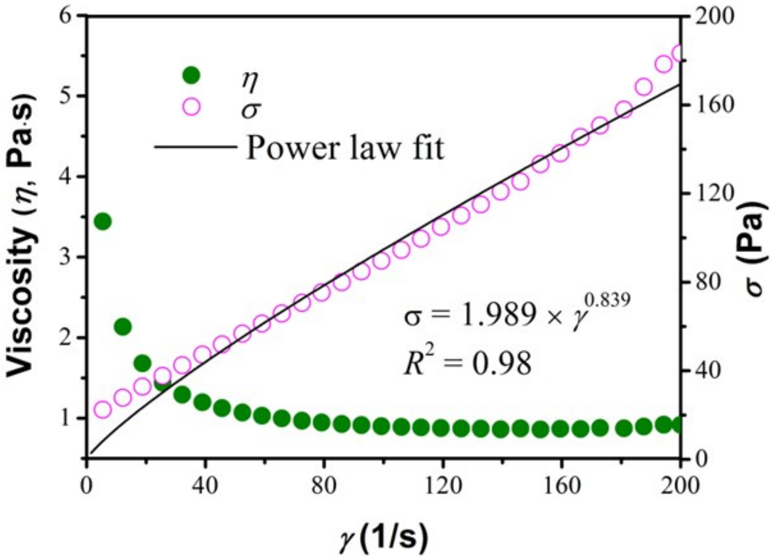

Fig. S2. Viscosity and shear stress with power law fit of a commercial petrolatum product.
